# Exploring Chromosomal Polymorphism and Evolutionary Implications in *Rineloricaria lanceolata* (Günther, 1868) (Siluriformes: Loricariidae): Insights from Meiotic Behavior and Phylogenetic Analysis

**DOI:** 10.3390/biology13090708

**Published:** 2024-09-10

**Authors:** Vanessa Isabel Batista de Morais, Juliane Vida Lemos de Oliveira, Alessio Alesci, Mara Cristina de Almeida, Roberto Ferreira Artoni

**Affiliations:** 1 Postgraduate Programme in Evolutionary Biology, State University of Ponta Grossa, Ponta Grossa 84030-900, PR, Brazil; vanessaisabelbm@gmail.com (V.I.B.d.M.); juliane.lemos.vida@hotmail.com (J.V.L.d.O.); 2 Department of Chemical, Biological, Pharmaceutical and Environmental Sciences, University of Messina, 98166 Messina, Italy; 3 Department of Structural and Molecular Biology and Genetic, State University of Ponta Grossa, Ponta Grossa 84030-900, PR, Brazil; almeidamara@uol.com.br

**Keywords:** chromosomal rearrangements, Robertsonian translocation, meiosis, rDNA, repetitive DNA

## Abstract

**Simple Summary:**

The genus *Rineloricaria* of Neotropical armored catfish has several species with chromosomal polymorphism, which is where individuals of the same population have different numbers of chromosomes and chromosome structures. This characteristic affects how these fish reproduce and adapt, but we do not fully understand its role in the evolution of this fish group. This study looks at *Rineloricaria lanceolata*, a species known for its chromosome polymorphism. It aims to understand how these different karyotypes arise and how individuals with different karyotypes can still produce offspring even when there is an imbalance in gamete generation. We used one individual as a model to find out how the karyotype was created. It turned out that two chromosomes from different pairs fused together to form a third larger chromosome. This resulted in an odd number of chromosomes, which led to different combinations of gametes being formed in meiosis because of how the chromosomes paired.

**Abstract:**

Chromosomal polymorphism is a significant aspect of population genetics, influencing the adaptation and evolution of species. In *Rineloricaria lanceolata*, a Neotropical fish species, chromosomal polymorphism has been observed, yet the underlying mechanisms and evolutionary implications remain poorly understood. This article aims to investigate the chromosomal polymorphism in *Rineloricaria lanceolata*, focusing on elucidating the meiotic behavior of karyotypic variants and tracing the phylogenetic origins of this polymorphism within the genus. By employing molecular markers and cytogenetic techniques, we aim to uncover the mechanisms driving chromosomal rearrangements and their potential role in speciation and adaptation. Understanding the genetic basis of chromosomal polymorphism in *R. lanceolata* not only contributes to our knowledge of species evolution but also holds implications for the conservation of genetic diversity within this vulnerable group of Neotropical fishes.

## 1. Introduction

Chromosomal polymorphism is a population attribute adhering to Ford’s definition (1940), “The occurrence together in the same habitat of two or more discontinuous forms, or phases, of a species in such proportions that the rarest of them cannot be maintained merely by recurrent mutation”. This can involve both the number and structure of chromosomes. Such variations may occur naturally and have implications for the adaptation and reproduction of organisms.

Polymorphism-generated variation can enhance genetic diversity, which serves as the raw material for evolution. In response to environmental changes, certain karyotypic variants may confer adaptive advantages, enabling individuals carrying them to achieve greater reproductive success and pass on their genes more frequently to the next generation. Conversely, karyotypic polymorphism can lead to segregation issues in gamete formation, resulting in offspring with genetic anomalies, akin to well-known chromosomal syndromes in humans (for a review dataset in OMIM, 2023).

Teleosts are widely distributed globally and extremely diverse, with over 30,000 known species [1]. Reports of chromosomal polymorphisms in this vertebrate group are abundant [2,3,4,5,6]. The three-spined stickleback (*Gasterosteus aculeatus*, Linnaeus, 1758) is a notable example, exhibiting chromosomal polymorphism with variations in chromosome number and sex determination systems among different populations. These variations have significant implications for the adaptation and evolution of this species in various aquatic environments [7]. Salmonids, representing a family of ecologically and economically important fishes, are well known for their broad karyotypic variation due to chromosomal polymorphisms [8]. Recent genomic studies have aimed to link chromosomal variations to adaptation, particularly in Atlantic salmon (*Salmo salar*). A variant chromosomal translocation is strongly correlated with temperature in a population of *S. salar* in Canada [9]. This suggests that Robertsonian polymorphisms play a role in the local adaptation of Atlantic salmon to specific environments.

An interesting group of Neotropical fishes, the *Rineloricaria* Bleeker, 1862, comprises 71 valid species [10], with polymorphic and non-polymorphic populations regarding karyotypic formulae in various species [11]. These small armored catfishes form population demes in small streams, and some are rheophilic species associated with fast-flowing streams [12], widely distributed in South American rivers [13,14]. Despite being considered a diverse group with complex taxonomy, molecular studies have suggested they form a monophyletic group (Figures S1 and S2) [15,16]. Several species/populations have been characterized with karyotypic variation attributed to Robertsonian structural rearrangements [17,18,19,20]. Furthermore, this karyotypic variation is not fully elucidated, with no investigation conducted at the level of meiotic chromosomes or understood within the phylogeny of *Rineloricaria* [16].

The origin and mechanisms involved in karyotypic variation in *Rineloricaria* remain an open question. The use of molecular markers at the chromosomal level, especially through in situ localization of repetitive sequences, has indicated the occurrence of centric fusions [19,21,22]. Rosa et al. [19] identified the occurrence of Interstitial Telomeric Sites (ITSs) associated with the location of 5S rDNA on chromosomes of *R. lima*. Based on these findings, the authors suggest that ribosomal DNA could play a role as a breakpoint facilitator for chromosomal fusion. Similar evidence was found in *R. pentamaculata* (Langeani & de Araujo, 1994) [21], additionally verifying the enrichment of these chromosomal regions with microsatellites (CA)n and (GA)n [22]. It has recently been demonstrated that in *R. latirostris* (Boulenger, 1990), besides the presence of rDNA sequences at possible chromosomal breakpoints, A-/G-rich microsatellites and sequences of the hAT retrotransposon are also present in these chromosomal sites [23].

Studying these populations with chromosomal polymorphisms contributes to a better understanding of the genetics and evolution of species, as well as the conservation of genetic diversity in vulnerable groups. Thus, our objective is to deepen knowledge about the chromosomal polymorphism described for *R. lanceolata*, especially regarding the meiotic behavior of a karyotypic variant and the phylogenetic origins of this feature in the genus.

## 2. Materials and Methods

### 2.1. Ethics Statement

Fish collections were authorized by Chico Mendes Institute for Biodiversity Conservation (ICMBIO—SISBIO—license number 15115-1), and the experimental procedures were approved by the Ethics Committee on the use of the Animals for Research at UEPG (CEUA Process number 0769342/2021). The use of the genetic data in this study was authorized by the National System for the Management of Genetic Heritage and Associated Traditional Knowledge (SISGEN number A6F96AE).

### 2.2. Sampling

**Figure 1:**
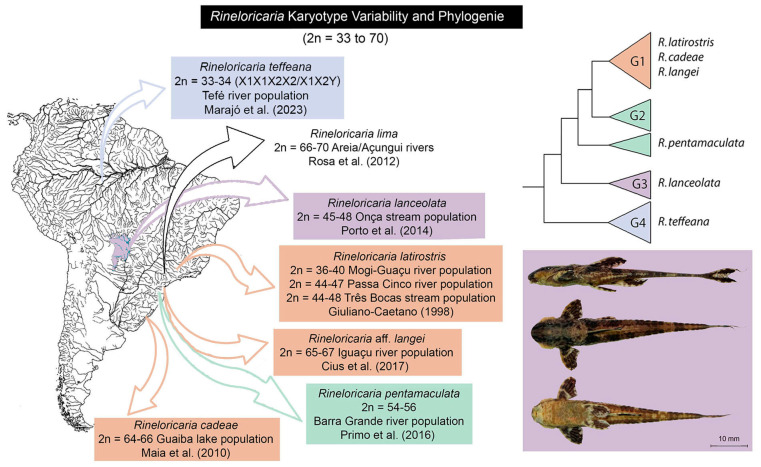
Chromosomal polymorphisms are described in populations of the genus *Rineloricaria* in different locations in Brazil [17,19,20,21,24,25,26].

Six individuals (three females and three males) of *Rineloricaria lanceolata* from the Onça stream, a tributary of the Taquari river (Coxim, MS, Brazil: 18°30′23.9″ S; 54°40′39.3″ W), were analyzed (Figure 1, in detail). All individuals were identified by morphological and barcode DNA criteria, and voucher specimens were deposited in the fish collections of the Museu de História Natural Capão da Imbuia, Curitiba, PR, Brazil (MHNCI), under the number MHNCI 12861.

### 2.3. Chromosome Preparations and DNA Extraction

Mitotic chromosomes were obtained from the incomplete regeneration of the animals’ dorsal fin, following the protocol by Kalous et al. [27]. The meiosis of a male individual was performed without prior application of intraperitoneal colchicine according to Bertollo et al. [28]. Images were captured with an AXIOCAM MRm camera in grayscale and a Zeiss AXIO IMAGER A2 microscope (Carl Zeiss, Oberkochen, Germany), then processed with ZEN PRO 2011, and the karyotypes were assembled using the Adobe Photoshop CC demo version (Adobe Software, San Jose, CA, USA). For each animal, at least 30 metaphase spreads were examined to validate the 2n and karyotype.

To characterize meiotic segregation, 30 cells were counted in meiosis II from a male individual with 2n = 47 chromosomes. The Chi-square test (α = 0.05) was performed to establish whether the haplotypes had the same frequency of formation in meiosis (H0) or some deviation favoring a certain type of segregation (H1). Chromosomes were classified as metacentric (m), submetacentric (sm), subtelocentric (st), or acrocentric (a) according to arm ratios [29].

Genomic DNA was extracted from the liver of all species according to Sambrook [30]. DNA quality was analyzed on a 1% agarose gel electrophoresis and with a NanoVue^TM^ UV/Vis spectrophotometer (GE Healthcare, Chicago, IL, USA). Before the procedures, the animals were sacrificed with clove oil (Eugenol) overdoses following international best practice recommendations for the use of animals in research.

### 2.4. Whole-Genome Sequencing (WGS) and Satellite DNA Identification

Total DNA samples underwent next-generation sequencing (NGS) on the BGISEQ-500 platform (BGI Shenzhen Corporation, Shenzhen, China) (paired-end 2 × 100 pb configuration). Genomic coverage was approximately 0.7× considering the size of 1.56 Gb from the reference genome of *Rineloricaria parva*. The sequence referring to RlaSat17-1328 was deposited on the GenBank database under access number PP336777.

In silico analyses were conducted in PARAM Shavak High-Performance Computing (State University of Ponta Grossa HPC project—NAPI/BioInfo https://www.iaraucaria.pr.gov.br/napi-bioinformatica/ (accessed on 23 July 2024)). Pre-processing of raw data was initially performed with Trimmomatic [31] for cropping and quality filtering of reads of Q > 20, which were then checked through FastQC reports. The files containing a sampling of library sequences were generated using the command “rexp_prepare.py” and submitted to the Galaxy platform 2024 update (https://repeatexplorer-elixir.cerit-sc.cz/galaxy (accessed on 25 July 2024)) for clustering and repetitive DNA characterization on RepeatExplorer2, TAREAN pipelines [32]. Mining was completed according to the SatMiner protocol 2022 update [33]; filtering of the initial satellites encountered was conducted using the software DeconSeq 2015 update [34], and the remaining sequences passed through a new RepeatExplorer clustering. The iteration of filtering and clustering occurred until no more satellites were found.

Further annotation of satDNAs was conducted using Geneious 2023.2 (https://www.geneious.com (accessed on 26 July 2024)) and Dimerator (DiaCarta) software 2.0 to search and remove homolog sites of multigene families. All remaining satDNAs were then characterized into variants, families, and superfamilies and named based on Ruiz-Ruano et al. [33]: sequences with at least 95% similarity were considered the same satellite; sequences from 80 to 95% similarity and similar size were considered families or variants; and groups with 50% or more similarity were classified as superfamilies. Afterwards, RepeatMasker [35] was used with the CrossMatch option and the Kimura 2-parameter model to estimate the abundance and divergence of each satellite in the genome.

Manual analysis of a clustering table generated by the RepeatExplorer protocol and TAREAN reports was performed to select satDNA sequences, of which RlaSat17-1328 was identified as a marker of interest for comparison with the 5S marker due to its labeling pattern in a single bivalent chromosome. Subsequently, primers were designed aiming at the development of probes for chromosomal mapping by fluorescence in situ hybridization (FISH) using Geneious 2023.2 and evaluated using the Multiple Primer Analyzer tool (Thermo Fisher Scientific, Waltham, MA, USA).

### 2.5. Chromosome Banding and FISH

The FISH procedure was conducted according to Pinkel et al. [36], under high stringency conditions, employing a 5S rDNA probe cloned from *Leporinus* [37] labeled with digoxigenin-11-dUTP and detection using anti-digoxigenin rhodamine (Roche, Manheim, Germany). Double FISH slides were prepared following the protocol with minimal adjustments, using probes for 5S rDNA and Sat17, respectively, labeled with Atto550-dUTP (green) and Atto488-dUTP (red) through Nick Translation (Jena Biosciences, Jena, Germany).

For double FISH, slides were incubated for one hour at 60 °C, followed by treatment with 0.005% pepsin at 37 °C for 5 min. Chromosomes were then denatured in 70% formamide/2 × SSC at 72 °C for 3 min. Simultaneously, probes were heated at 85 °C for 10 min and then cooled to 4 °C for 2 min. Hybridization mixes were composed of 50 ng of labeled DNAs, 50% formamide, 2 × SSC, 10% dextran sulfate, and Denhardt’s solution (pH = 7.0), making a total volume of 20 µL. Hybridization took place overnight in a dark chamber at 37 °C. On the second day of the protocol, slides were washed for 5 min in 1 × SSC at 65 °C and in 4 × SSC/Tween at room temperature, followed by a 1 min wash in 1 × PBS. After dehydration in an alcohol series, slides were mounted with DAPI and Vectashield (Vector Laboratories, Burlingame, CA, USA).

## 3. Results

The karyotype of six individuals of *R. lanceolata*, one of the polymorphic species of the genus (Figure 1), was mounted for identification of cytotype variation. The diploid number ranged from 2n = 45, 46, and 47 chromosomes, with FN varying between 48 and 52 (Table 1). All individuals had different cytotypes. Three individuals presented a diploid number of 2n = 47 chromosomes, and among these, three different cytotypes were determined: Cytotype A (2m + 2sm + 2st + 41a, NF = 51); Cytotype B (4m + 1sm + 2st + 40a, NF = 52); and Cytotype C (2m + 2sm + 2st + 41a, NF = 51). In Cytotype B, the presence of a large submetacentric chromosome relative to the chromosomes of the complement that had not yet been described for the different cytotypes of the species was observed, possibly representing a new chromosomal variation. Two individuals had a diploid number of 2n = 46 with distinct cytotypes: Cytotype D (1m + 4sm + 2st + 39a, NF = 51) and Cytotype E (2m + 2sm + 2st + 40a, NF = 50). Only one individual presented a diploid number of 2n = 45 chromosomes with Cytotype F (2m + 1sm + 2st + 40a, NF = 48).

**Figure 2:**
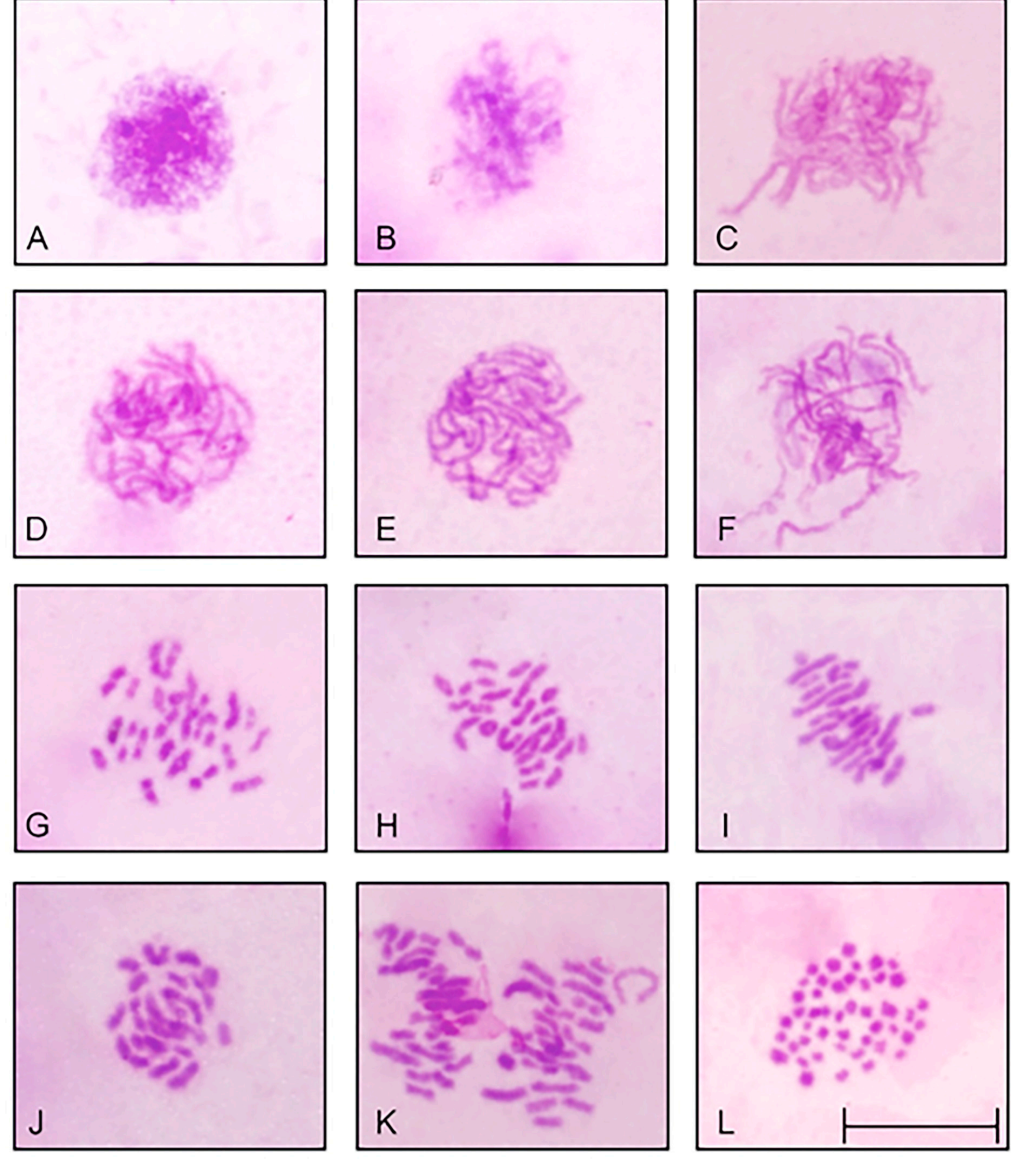
*Rineloricaria lanceolata* cells with conventional staining (Giemsa), 2n = 47. The dynamics of the gonadal cells of an adult male in meiotic division highlight the regularity of gamete formation, even though this individual has an odd diploid number. The karyotypic variants made possible by this process are shown in the idiogram in Figure 5. (**A**) Interphasis. (**B**) Zygotene. (**C**–**F**) Pachytene. (**G**) Diplotene. (**H**) Diacinesis. (**I**,**J**) Metaphasis I (two cells). (**K**) Metaphasis I. (**L**) Mitotic metaphasis. Scale: 10 µm.

**Figure 3:**
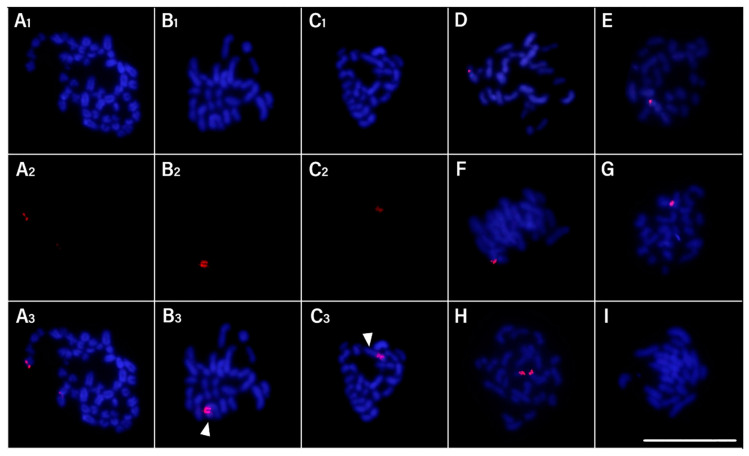
*Rineloricaria lanceolata* cells of the individual with 2n = 47 chromosomes with fluorescence in situ hybridization (FISH) using rDNA 5S probe (red) and DAPI counterstain. (**A_1_**–**A_3_**) Mitotic metaphasis. (**B_1_**–**B_3_**,**C_1_**–**C_3_**) Meiotic metaphasis. (**D**–**I**) All gamete segregation possibilities with emphasis on rearrangement-involved chromosomes during meiosis II are shown. Arrows: trivalent chromosomes paired in different positions during meiosis I. Scale: 10 µm.

**Figure 4:**
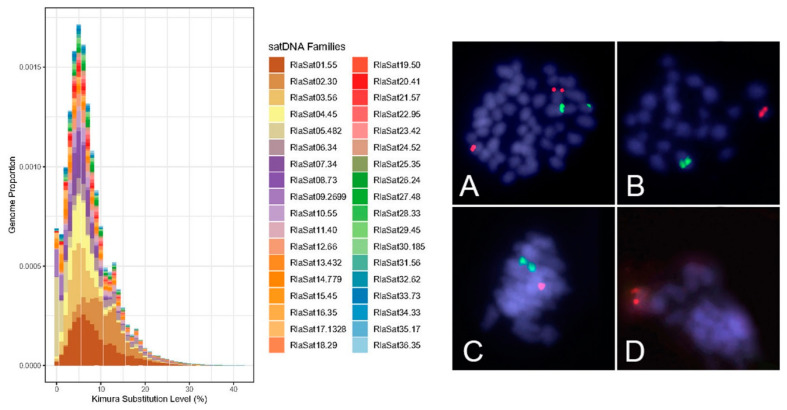
Satellite abundance and divergence profile data (**left**) and trivalent segregation highlighted by 5S probe (green) in contrast to bivalent conventional segregation shown with RlaSat17-1328 (red) in double FISH. (**A**) Mitotic metaphasis. (**B**) Meiotic metaphasis (n = 23) with the presence of submetacentric marked with 5S. (**C**) Meiotic metaphasis with both chromosomes marked with 5S. (**D**) Meiotic metaphasis with 5S marks absent. Note the satellite-marked chromosome is regularly present from (**B**–**D**) meiosis.

Slides were prepared with Giemsa staining (Figure 2) and fluorescence in situ hybridization (FISH) (Figure 3) for analysis of chromosome preparations from the fin and gonad of the Cytotype B individual (2n = 47). From the material, it was possible to locate the pairing of a trivalent chromosome in metaphasis, following the karyotypic association (22II + 1III). The FISH methodology also revealed that two of the chromosomes in the trivalent configuration were marked with the rDNA 5S probe and have distinct morphologies, one being submetacentric and one acrocentric, evidencing chromosomal rearrangement. The unconventional segregation of the trivalent chromosome when compared to a bivalent chromosome marked with the RlaSat17-1328 satellite probe is shown in Figure 4.

**Figure 5:**
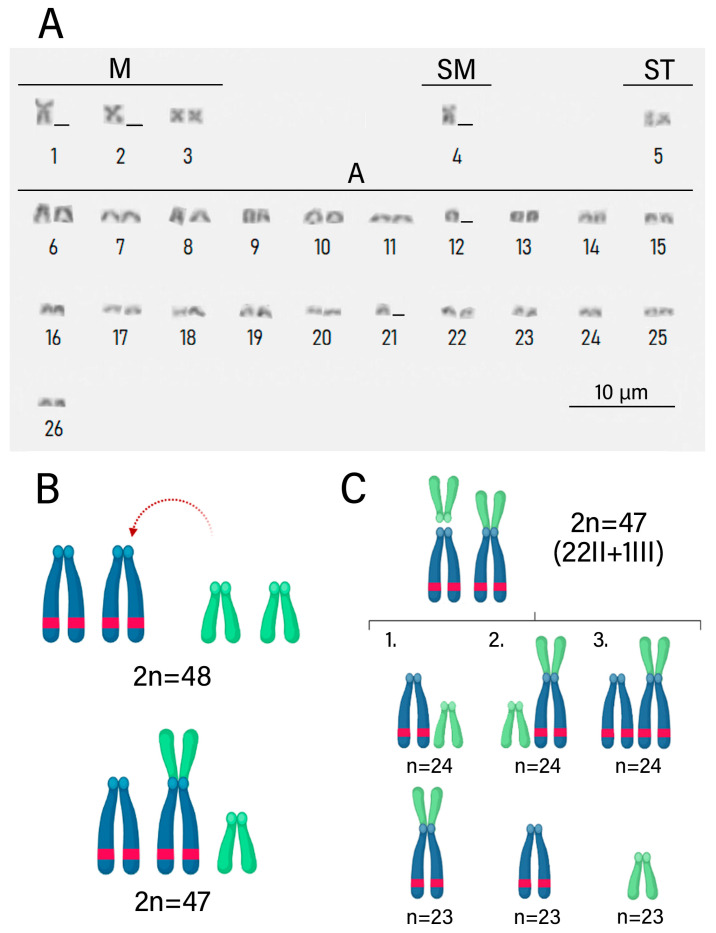
The new cytotype of *Rineloricaria lanceolata* originated from centric fusion and its consequences on meiosis. (**A**) Karyotype of B cytotype of a male individual, 2n = 47. (**B**) Origin of submetacentric chromosome through centric fusion. (**C**) Possibilities of gamete segregation during meiosis II of the chromosomes involved in the studied rearrangement.

Analysis of mitotic and meiotic metaphases from FISH slides was conducted to locate the chromosomes involved in rearrangement events and the segregation behavior of those chromosomes during meiosis (Figure 5). According to the scheme in Figure 5 and the meiotic metaphases in Figure 3, the chromosomal segregation comprised six different haplotypes, with half of the possibilities having n = 23 and the other half having n = 24 chromosomes, with the quantity of chromosomes with 5S markings ranging from 0 to 2. Table 2 presents the results of metaphase counting and the Chi-square test, which did not show a significant value (X^2^ = 2.8 < X^2^c = 11.07), supporting the null hypothesis that the haplotypes have the same frequency.

## 4. Discussion

Our study confirmed the presence of biarmed chromosomes in the predominantly acrocentric karyotype of *R. lanceolata*, reflecting a polymorphism where karyotypic variations are found in natural populations. The present description of a new variant (Cytotype B, with a new large submetacentric without a homolog) suggests the involvement of other chromosomes in Robertsonian rearrangements in this species. The diploid numbers 2n = 45, 46, and 47 found fixed in different individuals corroborate the findings of Porto et al. [20]. Differences were found in the karyotypic formulas given to the number of chromosome arms (NF, fundamental number), which reinforces the insight that chromosomal rearrangements are more complex in this species/population.

**Figure 6:**
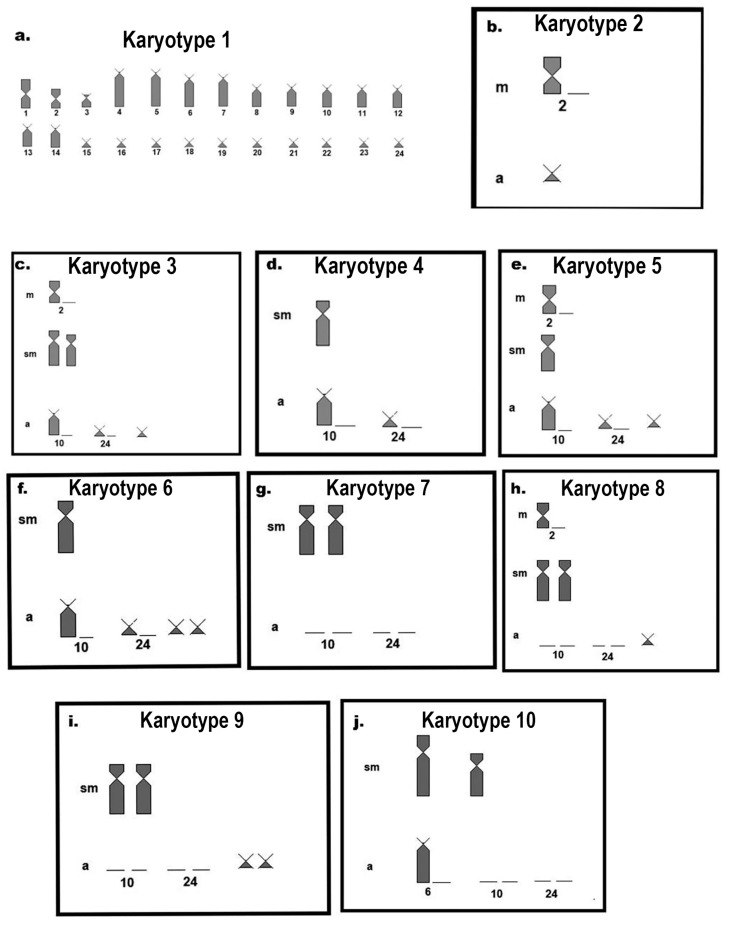
Cytotype ideograms found by [18]: (**a**) Cytotype 1 with 2n = 48, karyotypic formula: 4m + 2st + 42a, NF = 54;13; (**b**) variation found in Cytotype 2, with the absence of one of the homologous pairs of metacentric pair 2 and the presence of a small acrocentric chromosome; (**c**) in Cytotype 3, the absence of one of the homologous pairs of metacentric pair 2, the presence of a pair of medium submetacentric chromosomes, the absence of one homologous of acrocentric pairs 10 and 24, and the presence of a small acrocentric chromosome; (**d**) Cytotype 4, with the presence of a medium submetacentric chromosome and absence of one of the homologs of acrocentric pairs 10 and 24; (**e**) Cytotype 5, with the absence of one of the homologous pairs of metacentric pair 2, a medium submetacentric chromosome, absence of one of the homologous pairs of acrocentric pairs 10 and 24, and a small chromosome; (**f**) variation found in Cytotype 6, with a medium submetacentric chromosome, and absence of one of the homologous pairs of acrocentric pairs 10 and 24, and a pair of small acrocentric chromosomes; (**g**) Cytotype 7, a pair of medium submetacentric chromosomes, and absence of homologous pairs of acrocentric chromosomes 10 and 24; (**h**) Cytotype 8, absence of one of the homologous of metacentric pair 2, a pair of medium submetacentric chromosomes, absence of homologous pair of acrocentric chromosomes 10 and 24, and a small acrocentric chromosome; (**i**) Cytotype 9, a pair of medium submetacentric chromosomes, absence of homologous pairs of acrocentric chromosomes 10 and 24, and the presence of a pair of small acrocentric chromosomes; and (**j**) Cytotype 10, a large and a medium submetacentric chromosome, absence of one of the homologous pairs of acrocentric pair 10, and absence of homologous pairs 10 and 24.

The suppression of meiotic recombination attributed to chromosomal translocations is required as an important factor in karyotypic diversification and is reflected in speciation, given the infertility rate of hybrids in gamete formation [38]. On the other hand, at the intraspecific level, our results indicate that the occurrence of a large number of karyotypic variants in the population of *R. lanceolata* from Rio Coxim, Mato Grosso do Sul, Brazil (at least 10 cytotypes described, Figure 6), dismisses the occurrence of gamete drive. In the meiosis of the individual with Cytotype B, we were able to observe the occurrence of a trivalent chromosome in pachytene and follow it into metaphase I, as well as locate it in mitotic metaphases by fluorescent in situ hybridization with a 5S rDNA probe. FISH marking allowed us to propose that submetacentric chromosome n. 4 is derived from a Robertsonian rearrangement between two acrocentrics, one of which shares the same marking with 5S rDNA. Although not directly involved in the recombination site involved in the rearrangement, the 5S rDNA serves in this case as an important marker since it is shared only among chromosomes that have undergone this event.

The combination of possible gametes formed by Cytotype B (Figure 5B,C) and a hypothetical cross with a more conserved karyotype with predominantly acrocentric chromosomes would form karyotypic variants already described for this species in nature. These results suggest that populations of *Rineloricaria* experience karyotypic variation as balanced polymorphisms, as a strategy for maintaining genetic variability in populations with restricted gene flow. Similar karyotypic variation is often reported for other species of the genus *Rineloricaria*, such as in *R. cadeae* (Hensel, 1868) [26,39], *R. kronei* (Miranda-Ribeiro, 1911) [39,40], *R. latirostris* [17,21,23], *R. lima* (Kner, 1853) [19], *R. pentamaculata* [18,21], and *R. lanceolata* [20] (Figure 1).

The intrapopulation maintenance of karyotypic polymorphisms remains an intriguing question, given the inherent disadvantages of producing inviable gametes, especially aneuploids, in populations with a small number of individuals. The study by Buj et al. [41] with fish of the genus *Telestes* located in Croatia shares a similarity in that, similarly to *Rineloricaria*, they are found in water bodies whose hydrography has led to the formation of isolated populations with a low number of individuals. The study of genetic diversity in these species and populations showed that most populations had a high degree of diversity comparable to that found in large populations. One hypothesis to explain this event is described in the model proposed by Labar and Adami [42], which indicates the predominance of balancing selection in small populations and introduces the concept of “resistance to genetic drift”. While populations with a reduced number of individuals tend to maintain different traits, including mutations that may be slightly negative and decrease adaptive value, this decrease in the effect of genetic drift preserves genetic variability. The developed model also shows that, after reaching a point where there is a balance of a drift effect and the fixation of a considered positive trait, there is also a tendency to fix deleterious mutations, consequently decreasing the adaptive value of the population and returning to a state of resistance to genetic drift. This phenomenon could explain the occurrence of karyotypic polymorphisms in various species of *Rineloricaria*, given the characteristics of their populations.

The occurrence of karyotypic polymorphism attributed to Robertsonian events occurring in different species and populations of *Rineloricaria*, when confronted with the available phylogeny for these fishes, evokes the recurrence of homoplasies. All internal clades established by Covain et al. [16], represented by the letters G1, G2, G3, and G4 (Figures S1 and S2), present species with polymorphism and also with stable karyotypes. However, our understanding is that this is a bias caused by the hard interpretation from different areas of science. Even though it is a variable character at the population level and, as such, cannot be considered a good phylogenetic marker per se [43], the occurrence of karyotypic polymorphism in this genus requires a more parsimonious interpretation. We hypothesize that the underlying mechanisms of karyotypic instability (Robertsonian translocations) are ancestral in *Rineloricaria* and that, in certain situations, they are activated or deactivated.

Some authors have sought to attribute the breakpoints and centric fusions in different species of these fishes, but there is no consensus on the effective participation of moderately repetitive DNA such as ribosomal 5S [19,21,23] and 18S [21], heterochromatin rich in satellite DNA or telomeric sequences [20,22], or even the participation of pseudogenes and transposons [21,23]. Given this open question, we think that the diversifying mechanism (hot spots of chromosomal recombination by translocation), active or not, is what should be understood as the phylogenetic trait in the case of *Rineloricaria*.

Even for the most emblematic example of karyotypic evolution mediated by centric fusions, the case of the muntjac (Cervidae), it is still not fully elucidated in its origins and molecular mechanisms. Recently, a significant advancement was made by Yin et al. [44], in understanding the impact of centric fusions on the 3D chromatin architecture and the underlying molecular mechanisms of these rearrangements in muntjac deer. These authors suggest, based on high-density genomes associated with Hi-C mappings, that the centric fusion rearrangements experienced in different muntjac species are conserved (TADs, topologically associated domains) and have been facilitated by the presence of a complex repeat structure that led to illegitimate recombination between non-homologous chromosomes and, consequently, recurrent chromosomal fusions

In *Rineloricaria*, it is imperious to investigate the chromosomes or segments involved in rearrangements through chromosome painting, as well as to unravel the molecular and, perhaps, epigenetic mechanisms involved. The importance of chromosomal territories (TADs) in the nucleus due to the eventual proximity of the chromosomes involved in such translocations is not ruled out here. The fact is that this is a fascinating scenario for understanding both the maintenance of these polymorphic populations in nature and the adaptive value of different cytotypes.

## 5. Conclusions

In summary, our study on karyotypic variations in *Rineloricaria lanceolata* reveals the complexity of karyotypic evolution in this species. Robertsonian rearrangements play a fundamental role in generating karyotypic diversity and maintaining balanced polymorphisms within these populations. The preservation of genetic variability, despite potential disadvantages in producing inviable gametes, suggests the operation of balancing selection in small populations. Further investigation into the molecular mechanisms involved is essential for fully understanding karyotypic evolution in *Rineloricaria* and calls for caution in its phylogenetic interpretation.

## Figures and Tables

**Table 1:** Cytogenetic data found for study individuals.

Cytotype	2n	Karyotype Formula	FN ^1^
A	47	2m + 2sm+ 2st + 41a	51
B	47	4m + 1sm + 2st + 40a	52
C	47	2m + 2sm + 2st + 41a	51
D	46	1m + 4sm + 2st + 39a	51
E	46	2m + 2sm + 2st + 40a	50
F	45	2m + 1sm + 2st + 40a	48

^1^ FN: fundamental number.

**Table 2:** Observed frequency of gamete segregation possibilities in meiosis II cells (n = 30) and Chi-square (α = 5%).

Segregation Type	Observed Frequency
1A	5
1B	3
2A	5
2B	4
3A	8
3B	5
Total	30
${X^{2}}_{calc}=2.8<{X^{2}}_{c}=11.07$	

X^2^calc: calculated Chi-square. X^2^c: tabled Chi-square.

## Data Availability

The use of the genetic data in this study was authorized by the National System for the Management of Genetic Heritage and Associated Traditional Knowledge (SISGEN number A6F96AE), with the sequence referring to RlaSat17-1328 deposited on the GenBank database under access number PP336777.

## Supplementary Materials

The following supporting information can be downloaded at https://www.mdpi.com/article/10.3390/biology13090708/s1, Figure S1: *Rineloricaria* genus relation between phylogeny as proposed by Covain et al. [16] and tables containing available cytogenetic information from species (1A–1D). Figure S2: Continuation of Figure S1.
