# Supplementary figures and images for: Exploring Chromosomal Polymorphism and Evolutionary Implications in Rineloricaria lanceolata (Günther, 1868) (Siluriformes: Loricariidae): Insights from Meiotic Behavior and Phylogenetic Analysis
Source: Biology (Basel). 2024 Sep 10;13(9):708. doi: 10.3390/biology13090708 (PMC11428316; doi:10.3390/biology13090708)

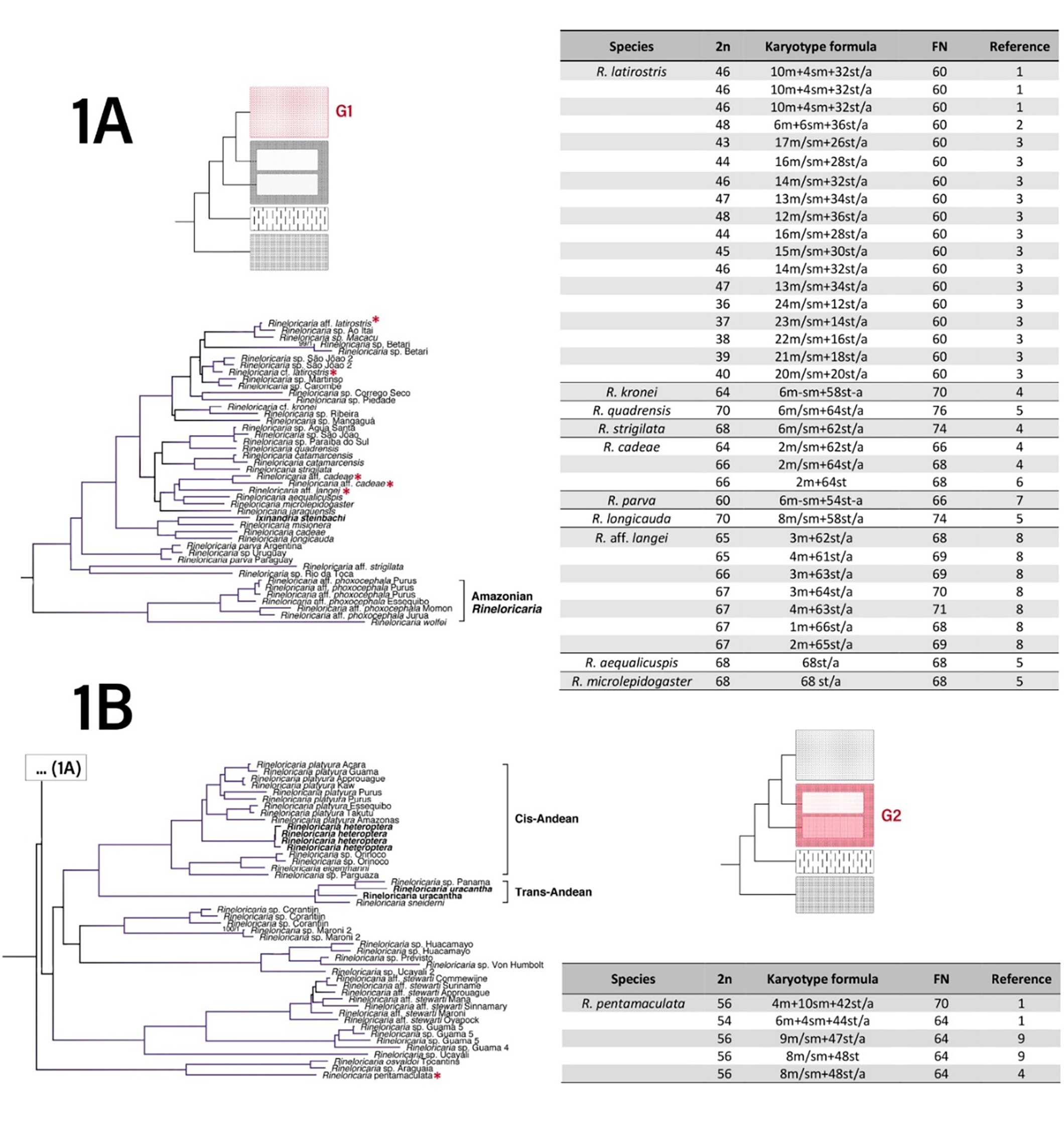

Supplement: Supplementary file 1 [file biology-13-00708-s001.zip › Figure S1.tif]

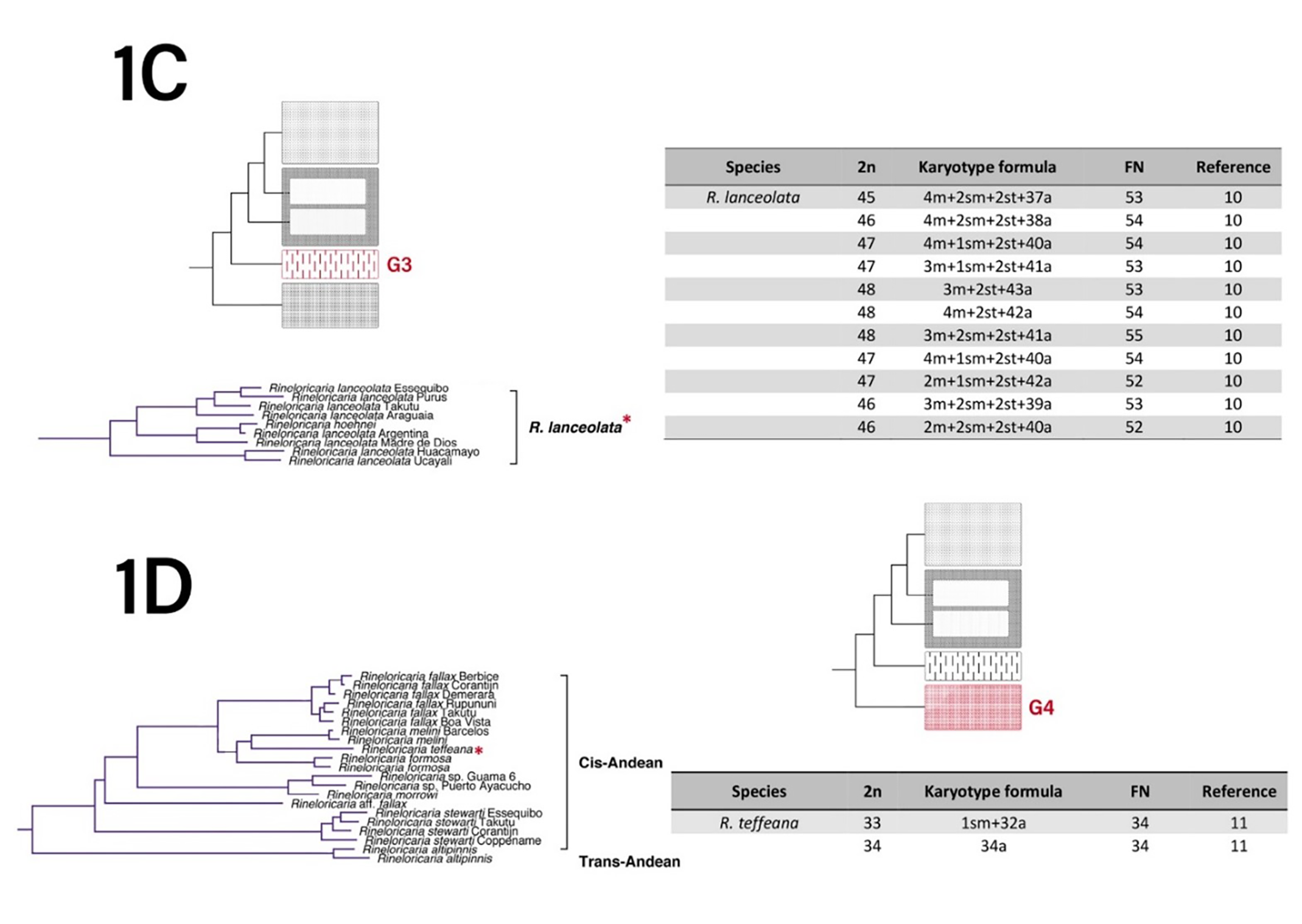

Supplement: Supplementary file 1 [file biology-13-00708-s001.zip › Figure S2.tif]
